# Gaze fixation parameters in basketball: examining the link to anxiety, self-talk, and emotional responses in free-throw shooting

**DOI:** 10.3389/fpsyg.2026.1730500

**Published:** 2026-02-11

**Authors:** Ioannis Ktistakis, Emmanouil Zacharakis, Gerasimos Terzis, Nikolaos Kostopoulos, Nektarios A. M. Stavrou

**Affiliations:** Faculty of Physical Education & Sport Science, National & Kapodistrian University of Athens, Athens, Greece

**Keywords:** anxiety, attentional focus, basketball, hand-eye coordination, quiet eye, self-talk

## Abstract

**Introduction:**

The quiet eye (QE) period is the interval of the final gaze fixation before the start of a decisive movement in each sport. QE is considered essential for successful performance in activities that require throwing or aiming at a target, such as basketball free-throw shooting. The purpose of the present study was two-fold, aiming to (a) compare gaze fixation parameters between successful and unsuccessful shots, and (b) investigate the relationships between gaze fixation variables, with eye-hand coordination, self-talk, anxiety, and emotion variables.

**Methods:**

Thirty-one basketball players participated in the Hellenic Women and Men U18 National Teams volunteered to participate in the study. Each participant completed ten free-throw shots while wearing the ET Vision system during the trials. The mean of the gaze variables value across these attempts was calculated. The participants filled in the Sport Anxiety Scale-2 based on how they usually feel, the Activation-Deactivation Adjective Checklist, and the Self-Talk Questionnaire based on how they felt before and during, respectively, the free-throw shots.

**Results:**

The results showed significant differences in gaze fixation parameters between successful and unsuccessful shots. There were significant negative correlations between concentration disruption and gaze fixation parameters. Positive and neutral correlations were observed between gaze parameters and instructional and motivational self-talk, respectively.

**Discussion:**

Successful free throws feature extended QE, onset, and longer fixation duration, and higher proximity of longer fixation to QE. Eye-hand coordination drills should be included in athletes’ physical training, as successful performance is a proactive, not a reactive, process. The application of psychological preparation programs and stress management techniques would help athletes to eliminate the negative effects of anxiety and disrupted concentration on their performance. These findings support a well-rounded basketball training approach that integrates technical instruction, visual strategies, and psychological skill development.

## Introduction

The duration of the final gaze fixation, known as Quiet Eye (QE), before performing a crucial movement has been studied across various sports. QE is essential for sport activities that require throwing or aiming at a target (Williams et al., 2002; Vickers et al., 2000). While the individual relationships among QE, competitive anxiety, and self-talk are well documented, the mechanisms by which these variables interact within a multidimensional framework remain scientifically and practically unclear. To clarify these interactions, energetic arousal can be operationalized as a heightened state of physiological activation preparing the body for action, while tense arousal is characterized by anxiety-inducing tension that may disrupt focus. Specifically, although prior research has largely treated physiological activation as a unidimensional construct, this oversimplifies key mechanisms, as energetic (activation) and tense arousal (tension) may exert distinct effects on QE characteristics such as onset, offset, longer fixation, and fixation frequency. Key studies, such as those pioneered by Vickers (1996), provide foundational insights into QE under pressure, and their findings highlight the importance of distinguishing between different forms of arousal in influencing QE outcomes. Thus, integrating these seminal works into the current exploration can enhance our understanding of these complex interactions. Furthermore, the attentional control theory links anxiety to working memory efficiency, but the mechanisms by which concentration disruption relates to QE timing, such as early versus late onset, offset, are unclear and have been only partially examined. The specific ways in which Self-Talk moderates these psychophysiological pathways are also not well understood and remain underexplored. It is not yet known whether self-talk preserves accuracy by extending QE duration or by stabilizing fixation onset in the presence of high tense arousal. The current research aims to clarify how self-talk relates to specific temporal features of visual attention (e.g., QE, longer fixation, onset, and offset duration) and to do so through a more comprehensive evaluation of participants’ psychological responses.

The QE period is defined as lasting at least 100 milliseconds and begins immediately before the final movement that determines skill execution in a given sport. For a fixation to qualify as QE, the gaze fixation must remain within 3 degrees of visual angle from the target. QE offset is identified when the gaze deviates from the target by more than 3 degrees for over 100 milliseconds (Vickers and Williams, 2007). During this final gaze fixation, motor programming is facilitated and attentional focus, a critical factor for successful skill completion, is enhanced (Vickers, 1996). Longer QE durations are generally associated with smoother and more effective execution of skill components (Causer et al., 2010). This extended focus further optimizes the direction, force, timing, and coordination of movements, ultimately resulting in improved performance (Mann et al., 2011). Empirical evidence indicates that a longer QE period positively contributes to the successful execution of free throws in basketball (Giancamilli et al., 2022). In addition to QE duration, the early onset of QE is another variable correlated with higher performance. Early onset provides a longer final focus period before hand movement and is linked to more successful attempts (Wilson et al., 2009). Research in golf (Vickers, 2012), ice hockey (Panchuk and Vickers, 2006), and shooting (Bayram et al., 2024; Janelle et al., 2000) demonstrates that elite athletes exhibit both earlier onset and longer duration of QE compared to lower-level athletes. Elite athletes display these characteristics more frequently than less skilled athletes, who do not achieve comparable performance levels (Causer et al., 2011b). More experienced athletes demonstrate fixations with greater accuracy, lasting 900 to 1,500 milliseconds, whereas amateur athletes typically exhibit gaze fixations lasting only 300 to 500 milliseconds (Rienhoff et al., 2015; Zhao et al., 2024).

Further to the above, it seems that both the athlete’s experience and success in performing an activity are closely linked with gaze characteristics. For instance, athletes with greater sport experience appear to perform with higher accuracy and speed (Vickers and Adolphe, 1997) and consistently exhibit fewer gaze fixations (Mann et al., 1998). In contrast, less skilled athletes display a greater number of shorter gaze fixations compared to their more skilled teammates (Williams et al., 2002). Previous studies indicate that, regardless of skill complexity or athlete level, QE duration is generally longer in successful attempts than in unsuccessful ones (Harle and Vickers, 2001; Janelle et al., 2000). Extended QE periods facilitate motor programming and enable optimal coordination of throwing speed, direction, and force for successful execution.

### Anxiety and QE parameters

Participation in sports activities and the execution of motor skills in competitive environments often occur under high-intensity conditions. These conditions are closely associated with anxiety, which can adversely affect athletic performance (Williams et al., 2002). In such settings, athletes must perform skills that continually challenge the boundaries of human capability. Empirical studies have shown that anxiety influences the QE period, a critical factor for tasks requiring precise aiming (Behan and Wilson, 2008; Shah et al., 2020). For instance, research using archery simulations has demonstrated that accurate aiming correlates with longer QE durations, but this duration is significantly reduced in the presence of anxiety (Behan and Wilson, 2008). Athletes experiencing elevated anxiety during competition typically exhibit shorter QE periods, which in turn impairs performance (Causer et al., 2011a). Evidence further indicates that anxiety-inducing situations can shorten the QE period and increase the frequency of brief gaze fixations (Williams and Elliott, 1999; Wilson et al., 2009). Moreover, extending the QE period and sustaining attentional focus during tasks that demand accuracy and aiming, even under heightened competitive anxiety, can improve performance and mitigate the detrimental effects of anxiety (Vine et al., 2011).

### Self-talk and quiet-eye performance

Μeta-analytic and systematic review evidence have provided substantial evidence that self-talk strategies in sport appear to be facilitative for athletes’ performance enhancement, facilitating also the learning process in the sport context (Tod et al., 2011), through cognition and anxiety regulation (Hatzigeorgiadis et al., 2011). In the context of fine motor skills and shooting accuracy, the self-talk is a critical determinant of success. The self-talk effects observed in different sport contexts and type of athletes highlighted the need to investigate the mechanisms underlying its effectiveness. Researchers examining the self-talk mechanisms have divided them into two broad clusters, that are the instructional and the motivational self-talk (Theodorakis et al., 2000). Based on relatively recent research findings, self-talk can improve the focus of attention (Bell and Hardy, 2009), whereas on the other hand reduces reaction time in cognitive attention tasks (Galanis et al., 2021), exerting a positive effect on gaze fixation parameters. Furthermore, sport task performance can be enhanced even under detrimental conditions such as external distractions (Galanis et al., 2017), ego depletion (Galanis et al., 2022a), and physical exertion (Galanis et al., 2022b). When athletes internalize directives, they sharpen their focus, which accelerates stimulus discrimination and decision-making. In more detail, instructional self-talk is positively linked to QE duration, extending the final fixation prior to execution. On the other hand, motivational self-talk aims primarily to regulate the physiological parameters (e.g., arousal) and effort. So, it seems that this type of self-talk is not closely linked to gaze fixation parameters, based on research findings.

### Attentional focus and gaze control

Attention and gaze focus are interdependent cognitive mechanisms that regulate perception, information processing, and motor skill execution. Contemporary neuroscience research indicates that these processes engage specific brain networks. These networks coordinate selective focus on stimuli through top-down processing and facilitate automatic detection of relevant changes via bottom-up processing (Eysenck et al., 2007). The dynamic interaction between these mechanisms is essential for complex activities such as reading, driving, and athletic performance.

The basketball free-throw is classified as a closed skill, with performance outcomes determined solely by the athlete’s ability. Smith and Kosslyn (2007) assert that concentration enables athletes to process relevant information for skill execution while filtering out distractions. In daily contexts, visual attention is shaped by both cognitive factors and sensory stimulation. Cognitive factors are linked to top-down processing, where information flows from higher to lower brain centers and is influenced by prior knowledge and experience. Sensory factors pertain to bottom-up processing, which involves the direct processing of sensory input leading to motor responses without observable feedback. Visual attention to sensory stimuli thus reflects an integration of bottom-up and top-down processes (Corbetta and Shulman, 2002).

Attentional Control Theory (ACT) posits that anxiety disrupts the balance between top-down and bottom-up attentional systems, resulting in impaired cognitive and motor performance. Multiple studies have examined the impact of anxiety on attentional concentration. Eysenck et al. (2007) found that anxiety reduces the effectiveness of the top-down system, leading to insufficient voluntary focus on the target, while simultaneously strengthening the bottom-up system and increasing sensitivity to distracting or negative stimuli. In tasks that demand advanced motor control, such as those performed by experienced athletes, both attentional focus and QE duration are critical (Vickers, 2007; Wulf et al., 2000).

### Eye-hand coordination

Coordination refers to the ability of the human organism to utilize different body parts in a smooth and effective manner. Motor coordination is a central topic in kinesiology, neuroscience, and physiology, with significant applications in athletic performance, education, and clinical rehabilitation. Coordination between the eyes and upper limbs constitutes a complex functional capability that links visual stimuli to motor responses. This process requires synchronized motor control of eye and hand movements, to enable actions such as catching and grasping objects (Grant, 2015) and accurately projecting objects toward targets (Rizzo et al., 2017). Effective eye-hand coordination ensures precise and synchronized hand movements, with gaze typically directed toward the target beforehand movement occurs (Chen and Tsai, 2015). Causer et al. (2014) investigated eye–hand coordination in the context of surgical knot-tying, finding that gaze fixations on task-relevant areas consistently preceded hand movement initiation. Superior performance was associated with longer fixations on critical areas, fewer overall fixations, faster knot-tying completion, and more rapid hand movements. The present study examines eye-hand coordination (EHC) as a critical factor in skill execution and the quality of athletic performance. In sports requiring precise timing, spatial perception, and rapid reaction, such as team sports, eye-limb coordination often determines outcomes. Wilson et al. (2013) reported that the QE period supports visuomotor control by letting trainees locate targets, plan movements, and adjust actions before execution, even in children with neuromuscular coordination disorders. A unique aspect of this study is its focus on the nuanced role of eye-hand coordination in both common and specialized athletic contexts, distinguishing it from the latest post-2018 meta-analyses which often generalize QE and anticipation characteristics across different sports. Our synthesis identifies a specific knowledge gap in the application of EHC strategies tailored for various athletic levels, from novices to elite performers. Effective eye-hand coordination requires longer fixations on key task areas and fewer overall fixations. This leads to faster and more efficient movements. Superior performance often ties to these patterns. In athletic tasks requiring precision and timing, eye-hand coordination is key. A key finding is that this coordination reliably directs gaze fixation toward the target beforehand movement. This facilitates seamless integration of visuomotor skills, essential for high performance. Specifically, the quiet eye period facilitates accurate movement planning and adjustment. This improvement in performance during the quiet eye period has been observed across a range of scenarios, from children with neuromuscular coordination disorders to elite athletes in competitive sports.

### Rationality and purpose of the study

Previous research indicates that in highly competitive situations, particularly towards the end of crucial basketball games with minimal score differences, free throws frequently determine the outcome (Sampaio and Janeira, 2003). Effective execution of this skill requires both technical proficiency and a strategic approach to gaze control, ensuring that the gaze remains steady in the face of competitive anxiety. This finding may stem from the fact that focusing attention on the details of the movement by increasing the QE period helps isolate the negative effects of anxiety.

The primary novelty of the present study was its integrative examination model of free-throw basketball performance. This holistic approach combines the study of athletes’ cognitive and emotional responses with their gaze fixation characteristics in high-level basketball players. Although QE is a well-documented predictor of success, this research examines the role of the longest fixation’s proximity to QE as a new metric. By analyzing the timing of this key period of visual information processing during the pre-shot routine, the study assesses whether specific cognitive strategies help anchor the movement plan more effectively. The study provides a detailed gaze analysis, including QE duration, onset, offset, longest fixation duration, and the number of fixations. This aims to offer more comprehensive motor and cognitive information. The approach is supported by using the Sport Anxiety Scale-2 (SAS-2) to measure athletes’ anxiety traits and the Activation Deactivation Adjective Checklist (ADACL) to evaluate arousal levels during free throws. Additionally, the study examines how different types of self-talk affect athletes’ attention. It tests whether instructional self-talk (focusing on technique) or motivational self-talk (focusing on effort or self-confidence) changes gaze fixation behaviors. The research seeks to determine if one self-talk type is more effective at positioning the longest fixation closer to the QE period, thus stabilizing motor control and improving elite performance. Addressing this gap is critical, since high-level athletes may process self-talk cues differently and possibly optimize neural efficiency by stabilizing gaze behavior earlier. It is worth noting that the link between motivational or instructional self-talk and gaze behavior parameters—especially onset, offset, and fixation duration—remains unexplored or only partly examined in high-level athletes. By combining these concepts with detailed gaze fixation parameters, the study aims to map the cognitive-motor pathway in free-throw basketball shots.

The purpose of the present study was two-fold. First, the study aimed to examine differences in gaze fixation parameters (QE, onset, offset, longer fixation, number of fixations, and longer fixation proximity to QE) between made and missed shots in high-level basketball players. Secondly, the study aimed to determine the relationship between gaze fixation parameters and emotional and cognitive responses during free-throw execution (self-talk, competitive anxiety, arousal, and eye-hand coordination). The following hypotheses have been examined: Significant differences will appear in QE, onset, offset, and longer fixation duration between made and missed free-throw shots, as well as in the number of fixations and proximity of longer fixation to QE. Furthermore, it was hypothesized that gaze fixation parameters would show positive correlations to self-talk types, arousal, and eye-hand coordination, except for the number of fixations. On the other hand, anxiety and concentration disruption would reveal positive correlations to the number of fixations, whereas negative correlations would reveal QE, onset, offset, and longer fixation duration.

## Method

### Participants

The sample size determination of the current study was based on the meta-analysis by Lebeau et al. (2016) which reported a moderate effect size (g = 0.58) for a within-subject performance, between the hit and missed frow throw shots. The power (1–*β*) was 0.80, and *α* = 0.05. The analysis indicated a minimum of 26 participants (Faul et al., 2007). A total of thirty-one (31) basketball players participated in the study, representing a significant cohort given the recruitment constraints for high-level athletes (Swann et al., 2015). The participants’ mean age of 17.52 years (SD = 0.51). Among them, sixteen (16) were members the Hellenic Women U18 National Team and the fifteen (15) were the Hellenic Men U18 National Team. The players had an average competitive experience of approximately ten (10) years (*M* = 9.48 years, SD = 2.23 years) and they have competed in nearly 500 games (*M* = 493.52 games, SD = 217.86 games). The average height of the participants was approximately 1,9 meters (*M* = 189.36 cm, SD = 11.41 cm). Most of the players were right-handed (30 players), with only one being left-handed. In total, the athletes took 310 free-throw shots, 213 of which were successful (68%), whereas 97 were missed (32%).

### Instrumentation

#### Eye tracker

The Eye Tracker is a portable device (ET Vision system—Applied Science Laboratories; Bedford, MA) composed of a pair of glasses (ET Vision optics) and a data-recording unit (ET Vision controller) secured at the participant’s waist with a strap. The glasses contain two internally mounted cameras, each capturing the movement of one eye at a frequency of 180 Hz using infrared light. A third camera, positioned at the front of the frame, records the environment or object the gaze is directed at. The controller connects to a computer via LAN (Ethernet cable) or WiFi, enabling the use of recording and data analysis software. An external camera, specifically the laptop’s built-in camera in this study, is connected and automatically synchronized with the ET Vision system. This camera is positioned to capture the complete movement of each participant during free-throw execution. The Eye Tracker, in conjunction with its software, records multiple data points. It identifies the precise location of gaze (gaze focus), the duration and frequency of gaze fixations, and quantifies saccadic movements by measuring amplitude, duration, and maximum speed. The system integrates images and video with gaze data, determines areas of interest, and detects dwell periods within these areas. A circular cursor, corresponding to 1° visual angle with a 4.5-mm lens, indicates gaze location in video scenes. The system provides a spatial accuracy of ±0.5° visual angle and 0.1° precision. Additionally, it records the frequency of involuntary blinks and measures pupil diameter.

The system used a Dell G15 laptop running Eye Vision (ASL) software. This allowed real-time viewing of gaze data on the laptop, which was then recorded for offline analysis. The ET Vision controller was connected to the laptop with a 10-meter FireWire cable, enabling participants to move almost normally. To limit distraction, the experimenter and laptop were positioned behind the participants. Additionally, an external camera (the laptop camera in this study) was also connected and automatically synchronized with the ET Vision system. This laptop camera was placed 3 meters to the right or left, depending on whether the shooter was right- or left-handed, and oriented perpendicularly to the shooting direction (in the sagittal plane). This setup captured the full free-throw action for offline analysis.

The ET Vision System used to evaluate several gaze-related variables. Each variable’s mean was calculated across ten free-throw attempts per participant. The total duration of the final gaze fixation on the basket (QE) was measured before the forearm–upper arm angle began to increase at the start of the shooting motion. In a typical free throw, this final fixation is often interrupted when the ball passes in front of the eyes, after which participants may briefly refixate on the target before beginning the shooting motion. In this study, QE duration comprised the initial fixation plus any subsequent shorter fixations after the interruption, provided they fell within the QE period before the arm angle increase. The onset phase refers to the period from the start of QE fixation (when the eyes focus steadily on the target) to the increase in elbow angle. This interval, a component of the overall QE (quiet eye) duration, is crucial for free-throw preparation and planning. The offset phase begins with forearm extension and ends with the conclusion of QE fixation. According to Vickers (2007), QE ends when the gaze fixation deviates by more than 3 degrees from the target for over 100 milliseconds. Thus, the total QE duration is the combined length of the onset and offset phases. Fixation frequency, calculated as total fixations divided by free-throw duration (fixations per second), reflects ocular stability (steady gaze) or restlessness (frequent shifts). Additionally, the study examined the longest fixation duration during the athlete’s free-throw preparation. It then assessed whether the sequence position of this gaze fixation, relative to the QE fixation, affected free-throw effectiveness. The analysis focused on the spatial and temporal relationship between these fixations and their impact on shooting accuracy.

#### Eye-hand coordination

Participants’ visuomotor coordination is assessed using the Photoelectric Rotary Pursuit Apparatus (Lafayette), which includes a stationary control unit and a pursuit rod with a photocell. An electronic timer records how long the rod contacts the moving target. The researcher adjusts target speed (1–100 cm/min), direction (clockwise or counterclockwise), and movement path (circular, triangular, or square) from the control unit. Participants try to keep the rod on the target as long as possible. After a familiarization trial, they complete three 30-s attempts. The total contact time, indicating visuomotor coordination, is the primary outcome.

To evaluate the psychological responses of the participants, the following instruments were used:

#### Activation-deactivation adjective check list

The Activation- deactivation Adjective Check List (ADACL; Thayer, 1989) is a multifactorial self-report instrument that evaluates the participant’s emotional state, and particularly the level of perceived activation. ADACL consists of twenty (20) items-adjectives which comprise four (4) factors: (a) activation, (b) tiredness, (c) tension, and (d) calmness. Each factor consists of five (5) items - adjectives, which are answered based on a four-point scale. The ADACL has shown acceptable indicators of reliability and construct validity (Ekkekakis et al., 2005; Thayer, 1989). The ADACL was selected to provide a multidimensional assessment of participants’ emotional responses through an ecological approach, despite ACT typically being examined under manipulated pressure. The ADACL distinguishes between energetic arousal (activation), associated with resource mobilization and readiness, and tense arousal (tension), linked to distress and potential attentional interference. This distinction is essential for QE research because it clarifies whether positive activation and negative tension exert differential effects on gaze behavior. Consequently, this approach enables a more nuanced understanding of how various types of arousal relate to gaze responses and athletic performance.

#### Self-Talk Questionnaire

The Self-Talk Questionnaire, developed by Zervas et al. (2007), is designed to evaluate the various forms of self-talk that athletes or trainees use to enhance their performance, particularly during exercises or training sessions. The questionnaire consists of eleven statements divided into two factors: motivational self-talk and instructional self-talk. Motivational self-talk includes seven statements (e.g., “During the free-throughs, I encouraged myself with positive self-talk”), while instructional self-talk comprises four statements (e.g., “During the free-throws, I talked to myself to correct my mistakes”). Participants respond to each statement using a 5-point Likert scale, with 1 indicating “Not at all” and 5 representing “Very much,” based on how they felt during the time of completion The questionnaire has demonstrated acceptable reliability and construct validity, as reported by Zervas et al. (2007).

#### Sport Anxiety Scale-2

The Sport Anxiety Scale-2 (SAS-2; Smith et al., 2006) is a multidimensional self-report instrument that evaluate athlete’s trait anxiety symptoms. The SAS-2 consisting of fifteen (15) items which comprise three factors: (a) somatic anxiety, (b) worry, and (c) concentration disruption. Each factor consists of five (5) items, and the participants respond to each item based on a four-point Likert scale ranging from 1 “Not at all” up to 4 “Very much.” SAS-2 have indicated acceptable construct validity and reliability. The SAS-2 stands out for measuring a specific concentration disruption factor that directly links to the attentional mechanisms governing QE, distinguishing it from other assessment tools in the field (e.g., Sport Competition Anxiety Test, Competitive State Anxiety Inventory-2). Based on attentional control theory, this approach tests whether trait anxiety chronically depletes working memory, impairing the top-down control needed for precise aiming (Eysenck et al., 2007). Evidence suggests that high-trait anxious individuals use inefficient gaze strategies and exhibit shorter QE durations due to attentional bias toward threat detection (Vickers and Williams, 2007; Wilson et al., 2009). The SAS-2 assesses inherent visuomotor vulnerabilities in the absence of external stress, highlighting how anxiety disrupts attentional regulation rather than merely causing physiological arousal.

### Procedure

The participants performed ten consecutive free throws from a standard distance of 4.57 m to a hoop set at a standard height of 3.05 m in an indoor facility. The selection of ten shots was based on prior research indicating that this number is usually sufficient to distinguish between “hits” (successful) and “misses” (unsuccessful) shots. A block of 10 shots has been used to evaluate differences in gaze characteristics between successful and unsuccessful shots and at it provides sufficient data to calculate the mean of QE duration and other gaze characteristics, while avoiding cognitive fatigue from longer trials (Harle and Vickers, 2001; Vickers, 1996; Vickers and Williams, 2007). Vine and Wilson (2010) also support that a block of 10 free-throw shots yields high data fidelity before a recalibration check is necessary.

The participants arrived at the court at least 45 min prior to the initiation of the research protocol. The athletes completed the SAS-2 based on how they usually feel when they compete and underwent the three trials of the eye-hand coordination evaluation. Participants then completed a 5-min warm-up. To minimize potential discomfort related to the equipment affecting free-throw execution, the athletes wore the eye-tracking glasses for 3 min prior to calibration to facilitate habituation. After the warm-up, the participants completed the ADACL immediately before the ten-free-throw execution based on how they felt at the time of completion. In the following, the calibration procedure was performed according to the protocol of the ET Vision System. Each participant underwent an auto-calibration process for the eye-tracking system, which involved sitting at a computer while wearing the ET glasses and fixating on a black dot at the center of a target displayed on the screen, according to the manual’s guidelines. When the calibration was successfully completed, the target disappeared from the screen. This automatic calibration process lasted up to 30 s, and all participants successfully completed the calibration. Participants then executed ten (10) consecutive free throws, self-paced, in accordance with standard FIBA regulations. Immediately after the free-throw attempts, the participants completed the Self-Talk Questionnaire, indicating the type of self-talk used during execution. The research protocol applied in the present study was approved by the Research Committee of Ethics and Bioethics of the Faculty of Physical Education & Sport Science of the National and Kapodistrian University of Athens (Approval Number: 1607/00-01-1900/24-1-2024).

### Statistical analysis

Data are presented as means and standard deviation (± SD). Preliminary analyses were conducted to screen for outliers and verify assumptions of normality (Shapiro–Wilk test) and linearity. Descriptive statistics (Mean SD) were calculated for the questionnaire subscales (SAS-2, ADACL, S-TQ) and gaze fixation variables (QE, onset, offset duration, fixation frequency). Pearson’s product moment correlation coefficient (r) was calculated to evaluate the relationship between the examined variables and examine the research hypotheses. The magnitude of the observed correlations was interpreted in accordance with Cohen’s (1988) standard guidelines for behavioral sciences as: (a) small: 0.10 to 0.29, (b) moderate: 0.30 to 0.49, and (c) strong: 0.50 and above. Paired samples t-test was used to detect differences between successful and non-successful shots in the examined variables. Significance was accepted at *p* ≤ 0.05 using a two-tailed test design. Statistical analyses were performed with SPSS version 26.0 software (SPSS Inc., Chicago, IL, USA).

## Results

### Data screening

Univariate distribution analyses were performed, and the assumption of normality was assessed prior to data analysis. The Shapiro–Wilk test showed no significant deviations from normality (*p* > 0.05) for the variables examined. Furthermore, Q-Q plots and analyses of skewness and kurtosis indicated values within the acceptable range of ±2.0 (George and Mallery, 2010). Therefore, the use of parametric tests, including the paired-samples t-test and Pearson’s correlation, was justified. No missing data were observed among participants.

### Made vs. missed free-throw shots differences

In Table 1, the means (M), standard deviations (SD), t-values, *p*-values, and effect size (Cohen’s d) for made and missed free throws based on gaze data are presented. The results indicated significant differences in both QE duration and onset duration between made and missed shots. Specifically, QE and onset durations were longer in made shots than in missed free throws. Additionally, there were significant differences in the number of fixations and in the “distance” between the longest fixation and the QE duration. The data showed that the longer fixation was closer to the QE in made shots (*M* = 1.54) compared to missed shots (*M* = 2.30).

**Table 1:** Means (M), standard deviations (SD), *t*-values, *p*-values and effect size (*d*) in made and missed free throws in gaze fixation parameters.

Gaze fixation parameters	Free throws		*t*-value	*p*	*d*
	Made	Missed			
	*M* (SD)	*M* (SD)			
QE duration	0.97 (0.61)	0.68 (0.43)	4.254	0.000	0.764
Onset duration	0.65 (0.46)	0.42 (0.30)	3.354	0.002	0.602
Offset duration	0.32 (0.29)	0.26 (0.31)	1.916	0.065	0.344
Fixations’ frequency	1.96 (0.52)	1.89 (0.68)	0.765	0.450	0.137
LF duration	1.20 (0.62)	1.18 (0.63)	0.127	0.900	0.023
Distance of LF to QE	1.54 (1.22)	2.30 (2.21)	−2.062	0.048	0.430

LF, Longer fixation; QE, Quiet-eye. QE, onset, offset and LF duration are provided in sec. Distance of LF to QE was measured on number of gaze fixations.

### Correlation analysis

Table 2 presents the correlations among eye-hand coordination, SAS-2 subscales, and gaze data. Significant positive correlations were observed between eye-hand coordination and QE duration, irrespective of shot outcome. In successful shots, eye-hand coordination was positively correlated with onset duration and negatively correlated with the distance between the longer fixation and the QE. Somatic anxiety was negatively correlated with offset duration in made shots. Worry with both QE duration and offset duration for both made and missed shots. Notably, there were interesting findings regarding concentration disruption and gaze variables. In made shots, concentration disruption exhibited negative correlations with QE duration, offset duration, onset duration, and longer fixation duration, and a positive correlation with the frequency of fixations.

**Table 2:** Gaze fixation parameters: correlations with eye-hand coordination, and Sport Anxiety Scale-2 subscales.

Gaze fixation parameters	Eye-hand coordination	Somatic anxiety	Worry	Concentration disruption
QE duration				
Made	0.45*	−0.31	−0.36*	−0.45*
Missed	0.42*	−0.14	−0.27	−0.25
Onset duration				
Made	0.38*	−0.12	−0.24	−0.37*
Missed	0.10	0.09	0.05	−0.14
Offset duration				
Made	0.35	−0.47**	−0.39*	−0.37*
Missed	0.48*	−0.28	−0.42*	−0.20
Fixations’ frequency				
Made	−0.10	0.21	0.13	0.29
Missed	−0.02	0.28	−0.00	0.36*
Longer fixation duration				
Made	0.37*	−0.21	−0.13	−0.39*
Missed	0.21	−0.16	−0.23	−0.24
Distance of LF to QE				
Made	−0.42*	−0.16	−0.03	0.07
Missed	−0.32	−0.34	−0.16	−0.21

*p < 0.05.

LF, longer fixation; QE, Quiet-eye.

Table 3 displays the correlations among the Self-Talk Questionnaire, the Activation-Deactivation Affective Checklist, and gaze data. Significant positive correlations were observed between instructional self-talk and both the duration of QE periods and the onset and offset timing of successful free-throw shots. Additionally, longer fixation durations were positively correlated with instructional self-talk. In contrast, significant negative correlations were identified between instructional self-talk and both the frequency of fixations and the distance between the longest fixation and the QE period. As the longest fixation approached the QE period, the levels of instructional self-talk increased. No significant correlations were identified between motivational self-talk and any gaze variables for either made or missed shots.

**Table 3:** Gaze fixation parameters: correlations with Self-Talk Questionnaire, Adjective-Deactivation Adjective Checklist subscales.

Gaze fixation parameters	Instructional self-talk	Motivational self-talk	Activation	Tension	Tiredness	Calmness
QE duration						
Made	0.45*	0.13	−0.08	−0.33	0.25	0.36*
Missed	0.32	0.01	−0.04	−0.49*	0.21	0.39*
Onset duration						
Made	0.35*	0.04	−0.04	−0.25	0.15	0.27
Missed	0.10	−0.10	−0.05	−0.36*	0.04	0.09
Offset duration						
Made	0.40*	0.20	−0.10	−0.31	0.30	0.34
Missed	0.34	0.10	−0.01	−0.33	0.27	0.45*
Fixations’ frequency						
Made	−0.46**	−0.19	−0.03	0.31	−0.03	−0.42*
Missed	−0.48**	−0.32	0.14	−0.04	−0.08	−0.11
Longer fixation duration						
Made	0.38*	0.12	−0.09	−0.29	0.18	0.30
Missed	−0.03	0.03	−0.14	−0.16	0.17	−0.01
Distance of LF to QE						
Made	−0.44*	0.06	0.06	0.28	−0.25	−0.44*
Missed	−0.42*	−0.12	0.13	0.00	−0.16	−0.13

*p < 0.05.

LF, longer fixation; QE, Quiet-eye.

## Discussion

### Interpretation and theoretical implications

The present study provided significant insights into the relationship between gaze parameters, psychological factors, and performance in basketball free throws. The results demonstrated clear differences in visual attention parameters between successful and missed shots, while simultaneously highlighting the critical importance of specific psychological variables in free-throw shot success.

The primary finding of this study is that free-throw success in basketball is strongly associated with longer QE duration. These findings confirm previous research showing that players sustain longer QE during successful attempts, suggesting that extended QE enables critical cognitive processing, allowing athletes to program shot parameters such as force, speed, and direction. Prior studies also propose that QE protects optimal motor strategies from interference (Klostermann, 2019; Mizusaki et al., 2025). In addition to the QE duration, in successful shots, the athletes indicated a closer proximity between the longest fixation to the QE (1.54 vs. 2.30) than in missed shots. The findings suggest that successful performance might depend not on the QE duration but on the QE architecture, indicating that visual stability proximity is an important factor, not just the final look. In other words, an early, stable anchoring, how quickly an athlete stabilizes his/her vision, might optimize the motor programming in closed skills, such as free-throw shots. However, the role of athletes should be taken into account, as high-level athletes participated in the present, which might indicate a different pattern compared to lower-level athletes.

A secondary main finding is that successful shots also have longer QE onset duration (650 ms) than missed shots (420 ms), underlining the importance of early and steady target focus for accuracy. Players with higher accuracy begin gaze fixation sooner, allowing extended cognitive processing and motor preparation. Although QE offset was longer in successful shots, this difference was not statistically significant, suggesting that the offset is not critical for accuracy. Based on onset and offset findings it seems that QE is not a simple fixation in the end of athlete’s execution, but a proactive preparation phase. Based on the onset differences, it seems that starting the fixation earlier provides an advantage to athlete’s in “preprogramming” even before the movement even starts, to adjust skills motor characteristics (i.e., force, trajectory). On the other hand, the gaze fixation after the movement initiation is less critical, as no differences appear between the made and missed shots. Τhe QE period serves mainly as feed-forward rather than as a feedback mechanism, helping the athlete in the preparation of the skill execution. In conclusion, an athlete should focus on the target quickly and eliminate possible intermediate fixations before the QE fixation.

Significant positive correlations were found between eye-hand coordination (EHC) and QE parameters. In successful shots, greater EHC was associated with longer QE duration, earlier QE onset, and longer gaze fixation. These findings suggest EHC is important for successful shot execution in a closed skill, such as free-throw shot. In other words, QE reflects not just a cognitive state, but also visuomotor integration. Players with increased EHC showed earlier QE onset. This suggests that EHC gives the motor system a longer period of stability, leading to more controlled movements, faster decision-making, and improved targeting accuracy. Enhancing eye-hand coordination may help athletes increase their QE duration and potentially improve shot performance. This highlights the importance of a dual-track training model to improve gaze fixation parameters. These parameters should be trained not only in isolated conditions to improve QE, but also in practical, real-world conditions to improve athlete coordination.

Somatic anxiety demonstrated a negative relation with offset duration during successful shots. Greater muscle tension corresponded to a shorter final gaze fixation phase and early disengagement from the free-throw process. However, somatic anxiety exhibited weak negative correlations with visual stability measures, including the ability to maintain a steady gaze, suggesting minimal influence on these aspects. In contrast, worry and, mainly, concentration disruption was consistently displayed, although with medium negative correlations with gaze-related variables. Specifically, worry was negatively related to QE, onset and offset duration. The attentional disruption contributes to more fragmented and less effective visual behavior, as evidenced by a series of brief fixations that do not provide sufficient time for necessary information processing. These findings align with previous research indicating that anxiety can reduce QE duration during free-throw performance under pressure (Wilson et al., 2009). Based on this detailed examination of anxiety symptoms, the relation of gaze fixation parameters showed that cognitive interference results in an increase of ineffective, short fixation, and a decrease of stable eye fixation. The concentration disruption is the primary reason for gaze fixation parameters disintegration, and not the level of somatic anxiety symptoms. While muscle tension (somatic anxiety) marginally shortens the QE offset, concentration disruption and worry fundamentally decompose the gaze architecture, leading to increased fixation frequency. This supports attentional control theory (Eysenck et al., 2007), suggesting that anxiety negatively affects working memory, leaving insufficient resources for the stable visual processing required for motor accuracy (Wilson et al., 2009). Psychological training interventions should not focus solely on physical symptom management through breathing techniques but also emphasize enhancing attentional focus through targeted cognitive-behavioral strategies, such as mental rehearsal. Prioritizing attention concentration may enable coaches and athletes to improve performance outcomes, particularly in high-stress performance environments.

Self-talk provides valuable insight into cognitive strategies associated with successful performance. Previous research findings have supported the self-talk’s significant role in QE duration; however, the current study offered more detailed insights by examining the instructional and motivational aspects of self-talk. Instructional self-talk showed significant relations with performance, whereas motivational self-talk did not. Instructional self-talk narrows attentional bandwidth, thereby lengthening QE and improving gaze fixation metrics. While motivational self-talk may enhance an athlete’s psychological profile, such as increased self-confidence, it does not consistently improve gaze fixation metrics. Instructional self-talk was positively related to QE, onset, offset, and the longest fixation duration during successful attempts, whereas negative relations were revealed to frequency fixation and the longest fixation proximity to QE. These findings suggest that instructional self-talk, including skill execution instructions, appears to be more effective than motivational self-talk in improving technical and attentional aspects, such as QE and gaze behavior, especially when precision matters. Providing instructions enables athletes to focus on essential technical elements, resulting in more stable and effective visual strategies.

Based on the results of the present study, it appears that instructional self-talk is a more effective cognitive pillar than motivational self-talk for managing and improving gaze fixation parameters. Instructional self-talk appears to be effective for refining perceptual-attentional control. It orients athletes to focus on the movement and task-relevant visual cues, preventing interference from irrelevant stimuli, either internal or external, resulting in more stable, earlier, and longer fixations (van Raalte et al., 1995; Perkos et al., 2002). Although motivational self-talk can enhance an athlete’s self-confidence, instructional self-talk (IST) primarily regulates temporal patterns of attention by increasing QE duration, initiating earlier onset, and decreasing the number of fixations. Through IST use, the athlete is compelled to focus on technical cues, facilitating the analysis of relevant visual information, leading to reduced fixation frequency, providing support to the self-focus hypothesis, based on which the athlete’s interest shifts to technical cues, simplifying and organizing the motor plan by supporting the free-throw execution without distraction (Sarig et al., 2023). Further to the above, it seems that IST influences the longest fixation sequential proximity to the QE period. In other words, IST not only extends focus by increasing QE and the longest fixation duration, but also, importantly, places the most critical period of information processing before movement initiation. The current findings are heavily based on cognitive-motor theory, indicating that instructional self-talk plays a crucial role by improving gaze fixation and merging self-talk with visual cues to improve free-throw motor execution and success.

Another addition to the current research was the evaluation of athletes’ emotional responses during free-throw execution. Examining participants’ emotional responses during free-throw execution indicated that calmness was the primary determining factor. Calmness was positively related to QE duration in all attempts, negatively related to fixation frequency, and the longest temporal fixation proximity to QE in made shots. These results indicate that calmness facilitates optimal gaze fixation and attentional focus. Athletes remaining calm enables them to maintain long, stable fixations on key elements of shot execution with minimal distraction, establishing a more stable visual behavior. This state allows athletes to focus on relevant visual cues and avoid distractions from negative personal concerns or environmental distractions, facilitating perceptual-motor planning and execution. Furthermore, calmness facilitates movement planning before skill initiation by prolonging QE duration, providing sufficient time for motor programming and possible error detection (Mann et al., 2011). Finally, a calm athlete experiences reduced cognitive load and fewer interfering thoughts, enabling automatic execution in well-learned skills, as expected in the current study, given the high level of experience among the participating athletes. This interpretation is supported by the psychological efficiency hypothesis (Vine et al., 2011; Wilson et al., 2009).

In contrast, tension revealed a negative relation with QE duration and missed shots onset duration. This finding is consistent with previous research supporting the notion that suboptimal tension has a deleterious effect on gaze fixation parameters, as evidenced by shorter and delayed QE periods. This finding aligns with the current study’s previous findings that negative emotions (e.g., anxiety) are negatively related to gaze parameters. Tension, as other negative emotions, appears to disrupt the visual-perceptual control process, leading to compromised attention and diminished motor control. These findings align with the theories of the psychological efficiency hypothesis (Vine and Wilson, 2011) and attentional control theory (Eysenck et al., 2007). Both theories propose that effective emotional regulation positively relates to attention and motor control during the performed activity, resulting in longer gaze fixation stability and fewer fixations due to less cognitive effort and a more efficient attentional system. Finally, activation and tiredness did not show significant relations to gaze response characteristics, possibly because of the non-competitive nature of the study. Activation and fatigue may exert less influence on gaze performance than in competitive environments, which might provide fruitful insights into the link between gaze fixation parameters and athletes’ emotional responses.

### Practical applications

The findings of the current study supported the need for athletes and coaches to apply a more holistic, multi-dimensional approach that integrates coordination drills and training. Sport psychologists are important for educating athletes in cognitive-behavioral strategies and emotional regulation. Central to these findings is the implementation of Quiet Eye Training (QET), which researchers and practitioners should move beyond mere QE duration toward refining the gaze fixation procedure, including early onset, and longer fixation importance. In other words, training protocols must emphasize early gaze fixation on the target to support motor preprogramming, and this should be applied in conjunction with eye-hand coordination drills. By training athletes to establish an early, steady focus, coaches can help them avoid being affected by internal or external interference, especially in competitive environments. The findings supported the importance of instructional over motivational self-talk. Based on that, coaches should clarify athletes’ technical instructions during skill execution, and sport psychologists should implement them in their psychological preparation programs. In practice, athletes should be able to effortlessly recall and apply brief, technical cues that align with critical visual phases of the performed task at hand. However, the importance of motivational self-talk should not be understated, as it may be linked to successful emotional regulation of concentration disruption and worry and, consequently, improve gaze fixation stability.

Importantly, to achieve a level of calmness that appeared important, coaches and sports psychologists should implement routines that combine breathing and mental training techniques. This would allow the athlete’s visual system to remain stable under pressure. Also, coaches should implement court-side drills in training environments that simulate high-demanding conditions, helping athletes be better prepared and reducing the impact on their gaze fixation parameters. A systematic psychological and technical evaluation of athletes in both training and competitive environments would yield valuable insights into athletes’ strengths and weaknesses, aiming to improve the qualitative and quantitative aspects of gaze fixation.

### Limitations and future directions

While the study revealed important practical and theoretical findings, certain limitations must be taken into consideration, which will formulate future research directions. First, the use of non-competitive field settings may not reflect the high environmental pressure, complexity, and physical demands encountered during real competition. Observing the measured variables in the actual competitive environment, using portable eye-tracking devices, would help to clarify the significant role of concentration disruption, contrary to the somatic anxiety, activation, and tiredness, which did not prove to be significant in the environment the current study took place. The use of highly experienced athletes may not represent lower-level or novice athletes, who might exhibit discrete gaze fixation patterns. Involving athletes of different levels would provide evidence of the early onset or the longer fixation proximity to QE’s usefulness as a universal predictor of successful performance. Additionally, choosing a closed skill allows a more attentive measurement of QE parameters; however, it limits the applicability of the results. Extending research to open skills such as jump shot or passing would provide a more comprehensive understanding of visual behavior related to basketball performance, and maybe the role of the motor preprogramming hypothesis. Finally, athletes’ psychological responses were based on self-reported instruments, which might have limited the capture of athletes’ cognitive and psychological status. The combination of heart rate variability, EEG, and eye-tracking would provide a more objective measure of the cognitive-motor theory discussed in the present study.

## Conclusions and key findings

Traditionally, Vickers (1996) research approached the QE concept simply as the final fixation on a target before movement initiation. The current study provides a more detailed approach from just the duration of the gaze fixation parameters to the structure and the sequence of them. First, the current study shed light in the sequential proximity of fixations, and mainly that of the longer fixation. The findings revealed that the time of athlete’s eye stabilization is crucial in successful execution of a skill, as the total duration. This study showed that in successful shots a shorter interval exhibit between the longest fixation and the QE (1.54 vs. 2.30), suggesting that visual stability functions as a proactive preparatory phase rather than a reactive process. In line with this finding, is the earlier onset in successful compared to the missed shots indicating that this part of the QE period functions as a feed-forward mechanism. This enables the athlete to preprogram kinetic parameters such as force and trajectory, based on which early fixation enables athlete to retrieve from the long-term memory the appropriate cognitive motor schema to appropriately respond to the needs of the task. Further, it is important to be mentioned that although eye-hand coordination is usually examined in the context of general motor task, its relation to gaze fixation parameters in closed-skill tasks, remains insufficient studied. Finally, the current findings should be interpreted with caution as the athlete’s or skill’s characteristics may affect several of the gaze fixation parameters before the initiation of the final movement.

An important part in the current study was the examination of possible relation between athletes’ psychological responses, either as a trait characteristic, or as a state one. Previous studies aiming to explore the role of negative emotions, approached anxiety as a unidimensional construct. The current study provided a more detailed approach examining the physiological (somatic anxiety) and the cognitive (worry, concentration disruption) aspects of anxiety. The findings supported the notion that concentration disruption is linked to a more profound negative effect on gaze fixation parameters, providing support to the attentional control theory (Eysenck et al., 2007) while the muscle tension marginally effects QE. Furthermore, this finding is supported by the processing efficiency theory which assumes that concentration disruption consumes working memory and hinder athletes from maintaining a stable gaze.

In line with the above, the findings showed that emotional regulation -calmness- might facilitate QE parameters, resulting in successful free-throw execution. Calmness reduces mental strain, enabling well-learned motor skills to operate automatically and thereby preserving the athlete’s psychological efficiency needed to perform in high-demanding athletic environments.

Another significant adding of the current study was that the study provides a more detailed understanding of the different types of self-talk (instructional, motivational) in gaze fixation patterns. Although self-talk is well-documented for athletes’ performance enhancement, the current study underlies the importance of the instructional self-talk. Instructional self-talk showed that lengthens gaze fixation parameters and diminishes fixation frequency. Although motivational self-talk does not appear to be related to ocular characteristics, it should be noted that the measurements were conducted under neutral, non-competitive conditions. In competitive environments, the impact of motivational self-talk may vary, as it has the potential to reduce the negative influence of external factors. Consequently, additional research is necessary.

In conclusion, based on the findings of the current study, should be noted that early initiation is also important as the duration of the QE. Instructional self-talk should be prioritized over motivation self-talk, to improve gaze fixation parameters. Managing concentration disruption and generally the cognitive elements of anxiety in contrast to the physiological elements of anxiety are more important in QE parameters improvement. The current study indicates that successful free-throw shooting results from a structured visual framework, which is reinforced by technical self-talk and successful emotional and cognitive regulation.

## Data Availability

The raw data supporting the conclusions of this article will be made available by the authors, without undue reservation.
